# The Base Rate Study: Developing Base Rates for Risk Factors and Indicators for Engagement in Violent Extremism†

**DOI:** 10.1111/1556-4029.14282

**Published:** 2020-01-30

**Authors:** Caitlin Clemmow, Sandy Schumann, Nadine L. Salman, Paul Gill

**Affiliations:** ^1^ Department of Security and Crime Science University College London London U.K

**Keywords:** base rates, terrorism, risk assessment, Prolific, unmatched count technique, threat assessment, online survey methods, violent extremism, lone‐actor terrorism

## Abstract

Improvements have been made in identifying the prevalence of risk factors/indicators for violent extremism. A consistent problem is the lack of base rates. *How* to develop base rates is of equal concern. This study has two aims: (i) compare two methods for developing base rates; the Unmatched Count Technique (UCT) and direct questioning, (ii) generate base rates in a general population sample and compare these to a sample of lone‐actor terrorists (n = 125). We surveyed 2108 subjects from the general population. Participants were recruited from an online access panel and randomly assigned to one of three conditions; direct survey, control, or UCT. Survey items were based on a lone‐actor terrorist codebook developed from the wider literature. Direct questioning was more suitable under our study conditions where UCT resulted in deflation effects. Comparing the base rates identified a number of significant differences: (i) lone‐actor terrorists demonstrated propensity indicators related to a cognitive susceptibility, and a crime‐ and/or violence‐supportive morality more often; the general sample demonstrated protective factors more often, (ii) lone‐actor terrorists demonstrated situational indicators related to a crime‐ and/or violence‐supportive morality more often, whereas the general sample experienced situational stressors more often, (iii) lone‐actor terrorists demonstrated indicators related to exposure to extremism more often. Results suggest there are measurable differences in the prevalence of risk factors between lone‐actor terrorists and the general population. However, no single factor “predicts” violent extremism. This bears implications for our understanding of the interrelation of risk and protective factors, and for the risk assessment of violent extremism.

Empiricism in terrorism studies is increasing 1. One systematic review of factors associated with violent extremism found 50 empirical articles 2. Studies typically cover areas concerning socio‐demographic characteristics, criminal history, religion and spirituality, work and education, personal experiences, attitudes and beliefs, relationships, mental health, motivation, radicalizing processes, and environmental factors 2. This empirical evolution spawned the development of a number of violent extremist risk assessment tools in the public domain including the Extremism Risk Guidance, Islamic Radicalization‐46, Identifying Vulnerable People, Multi‐Level Guidelines, Terrorist Radicalization Assessment Protocol, and the Violent Extremism Risk Assessment 3.

A consistent problem in both the study of violent extremism and the subsequent implementation of violent extremist risk assessment is that of base rates 4. There has been no attempt to explicitly measure how often these behaviors or experiences of interest occur in a nonextremist population. The same is largely true for general violent risk assessment research 5, however here, control group studies are much more prevalent. Control group studies are rare in violent extremist research. Desmarais et al.’s 2 systematic review found just six. Generalizing results from research designs lacking adequate control or comparison groups likely overpredict violent extremism. This problem is compounded when considering the relatively low occurrence of terrorism in the West 6. Although some factors are highly prevalent in some violent extremist samples, whether this finding is unique to violent extremists, or whether these risk factors are less, just as much, or more prevalent in the general population, is unclear. Addressing this gap in the literature offers important insights for the social scientific study of the causes of violent extremism. This drives the need for the development of base rates from a scientific perspective.

From a policy and practice perspective, generating base rates for predictors of violent extremism in a general population sample will help develop more rigorous putative risk and protective factors 7, increase transparency in the provision of evidence 8, reduce potential bias in decision‐making (9, however, see Ref. 10), improve risk communication 11, and allow for risk assessments based on Bayesian principles 12. Knowledge of base rates could also inform different forms of risk and threat assessment differently, as well as guide risk management and intervention (see Discussion).

The existing literature provides little to no guidance on *how* to develop base rates. Determining the prevalence rates of experiences, attitudes, or behavior that may be considered sensitive is challenging. In the context of this study, we focus on self‐report data to identify base rates using *direct* or *indirect* questioning, here the Unmatched Count Technique (UCT). Given the lack of previous research to draw upon, we undertake a test of both techniques. We then compare the base rate estimates of risk factors identified in a general population sample with those predetermined in a dataset of 125 lone‐actor terrorists 13, to specify risk factors that may be overestimated when only considering samples of violent extremists.

## Background

In the following section, we first discuss the risk indicator evidence base. Second, we describe research on violent extremists that employ some sort of control group. Lastly, we detail the rationale for undertaking a test of survey methods.

### Risk Indicators

Risk factors for violent extremism can serve as markers to inform the detection and disruption of terrorist threats. A systematic review showed that age, socioeconomic status, prior arrest, education, employment, relationship status, having a grievance, geographic locale, and type of geographic area, are factors associated with violent extremism 2. Other systematic reviews, rapid evidence assessments, and research syntheses report similarly 7, 14, 15, 16, 17. Some studies moved beyond distal risk factors and developed prevalence rates for a range of behavior‐based indicators 18. Further follow‐up studies conceptualized such risk factors and indicators as relating to propensity, situation, and exposure (for a full discussion see Refs. 13, 19, 20).

Propensity refers to developmentally relevant characteristics which may relate to a person’s predisposition for engaging in future offending. This is often conceptualized as the outcome of the radicalization process which has been modeled extensively 21, 22, 23, 24, 25, 26. Equally, pathway models of engagement in terrorism often refer to a radicalization phase 27, 28, 29, 30, 31, 32, 33. Such factors thought to influence violent extremism have also been examined empirically. These include national identity and attitudes 34 belonging and autonomy 35, religious attitudes, beliefs and ideologies 36 , religious identity, political attitudes, and suicidality 37, and other risk factors associated with radicalization 38.

Situational indicators relate to a person’s environment or context. These differ from propensity indicators in that they refer to a more proximal vulnerability. They include behaviors involved in attack planning and preparation, as well as behaviors related to operational security 18, 39, 40, 41, 42, 43, 44, 45, 46. Situational indicators may have important implications for risk assessment as they can signal the emergence and maintenance of the motivation to pursue terrorist violence 13, 19.

Exposure relates to encounters, online or offline, with people, places or settings which promote extremist violence or an extremist morality. Exposure may serve as a crude proxy measure for the prevalence of extremism in a general population. Equally, the extent to which the general population is exposed to or interacting with terrorism‐supportive people, narratives, or places, is of great interest. Here, exposure is operationalized with indicators related to network connectivity, such as interactions with other extremists, and leakage, that is, the extent to which someone communicates (directly or indirectly) an intent to commit violence.

The extent to which violent extremists operate within networks or in isolation has been researched extensively. For example, considering lone‐actor terrorists, researchers question “*how alone are lone actors?*,” with findings indicating that they may not be as “lone” as previously assumed 18, 40, 47, 48. Equally, the extent to which a range of violent offenders leak their intent has been examined empirically 18, 49, 50, 51, 52, 53, 54, 55, 56. This is often key to the threat assessment of these types of offenders. Therefore, we measure the extent to which a general population may have witnessed such behaviors. Hence, the present study examines risk factors relating to *propensity, situation,* and *exposure* in a general population. As previously stated, there has been no attempt (that the authors are aware of) to do so. However, some studies of violent extremists employ control or comparison groups.

### Comparing Violent Extremists and Control Groups

The findings of studies that employ control or comparison groups provide context for the results of the present study. Some studies compare different types of terrorists 57, or compare terrorists with nonviolent extremist offenders or subjects of concern 38, 58, 59. Others compare terrorists with analogous offenders like mass murderers 60, and some compare those with and without violent extremist attitudes 61. For the purpose of the present study, we outline the results of studies that compared those who engaged in terrorism or held attitudinal affinity with a violent extremist cause, with members of the general population.

First, some studies focus on socio‐demographic characteristics. The results are mixed. Altunbas 62 found U.K.‐based jihadist terrorists (*n* = 54) to be younger and more educated than the general population. Conversely, Costello et al. 63 surveyed 1034 youth and young adults in the United States and found that less education was associated with exposure to online extremism. Furthermore, Bartlett and Miller 64 compared terrorists to those who held extreme yet nonviolent beliefs. Terrorists were less likely to be employed and generally less educated.

Some studies focus on sociological and/or psychological aspects. For example, Bartlett and Miller 64 found no difference in terms of alienation, experiences of discrimination, and levels of religiosity between terrorists and nonviolent radicals. Pauwels and De Waele 65 looked at self‐reported right‐wing political violence among a sample of 2879 Flemish adolescents. Those who self‐reported conducting political violence were less socially integrated. Furthermore, Nussio 66 compared voluntary and nonvoluntary joiners of Colombian insurgent and paramilitary groups, arguing that the nonvoluntary joiners would have similar characteristics to nonjoiners. Despite similarities in demographic characteristics, the nonvoluntary joiners scored higher on three measures of sensation seeking (boredom susceptibility, disinhibition, and thrill and adventure‐seeking).

More complex research designs look at a range of influences. For example, Bhui et al. 67 found that those who scored higher on sympathies for violent extremism were older, more educated, suffered depressive symptoms, had problems with the police, and reported having something valuable stolen. Those who scored lower were less likely to have recently suffered the death of a close friend, relative, partner, spouse, child, or parent. They were also less likely to report interpersonal problems, a serious injury or illness, a major life stressor, and engaged in less nonviolent political activity.

Other studies have found higher rates of particular mental health disorders within terrorist samples compared to the societal base rate. These studies include schizophrenia and psychosis in Dutch foreign fighters 68, schizophrenia, autism and delusional disorder in lone‐actor terrorists 57, and subscale measures of psychopathic deviate, paranoid, depressive, schizophrenic, and hypomanic tendencies in Palestinian and Israeli terrorists 69. Other studies find lower rates of personality disorders and psychiatric illness compared to nonideologically inspired murderers 70. Dhumad et al. 71 compared 160 individuals convicted of terrorism, 65 convicted murderers, and 88 controls. Compared to the controls, terrorists were more likely to have had persistent childhood disobedience, a conduct disorder, and were less likely to have been treated harshly as a child. Compared to the murderers, terrorists were less likely to have an antisocial personality disorder, have had a family member murdered, or be easily provoked. 

Hence, there is some evidence to suggest that violent extremists differ in measurable ways from the general population, as well as from other types of offenders. Control group studies are key to developing an understanding of the behaviors and characteristics of a range of violent offenders. However, in terms of developing base rates, control group studies only measure and report on the limited selection of independent variables they employ. It would not be expected otherwise, as developing base rates has largely not been the purpose of this type of research. The present study therefore makes an important contribution to this literature as the first study to explicitly measure the extent to which risk factors for violent extremism occur in a general population sample. However, *how* to do so is equally of concern.

### Developing Base Rates

Risk factors for violent extremism involve experiences, attitudes, or behaviors that may be considered sensitive information to share. Hence, determining the prevalence rates of risk factors is challenging. One approach to capture the respective indicators is through self‐report data. In this context, direct questioning techniques require participants to answer a series of questions, directly. This includes questions relating to socially desirable concepts, such as voting or pro‐social attitudes, as well as questions relating to socially undesirable concepts, such as racism or homophobia. Directly self‐reported information, however, can be subject to a number of biases and errors, including underreporting socially undesirable items, overreporting socially desirable items, interviewer effects, bystander effects, and more 72, 73.

One factor in explaining the degree of misreporting in direct questioning is the mode of survey delivery (see Ref. 74 for a systematic review). Interviewer‐administered surveys, such as pencil‐and‐paper studies, or face‐to‐face interviews, can result in increased misreporting compared to self‐administered surveys. In fact, evidence suggests that self‐administered surveys may mitigate the extent of many of these biases or effects (see Ref. 73 for a review). Administering surveys online can mitigate these effects further by excluding the presence of an interviewer altogether 75. The results of studies that compare computer‐assisted self‐interview techniques to interviewer‐administered questionnaires equally suggest that limiting the presence of an interviewer may lessen the effects of biases 76, 77.

Online surveys have a number of additional advantages. These include a global reach, greater flexibility, speed and timeliness, the benefits of technological advances, convenience, ease of data entry and analysis, question diversity, low administration costs, ease of follow‐up, controlled sampling, larger sample sizes (that are easier to obtain), control of answer order, control of missing data (via required responses), and built in “go to” capabilities (e.g., “if yes go to question 2, if no skip to question 3”) to limit confusion and survey length 75. In a comparison of online, anonymous, self‐administered, and interviewer‐administered surveys, the most effective mode of delivery was found to be an anonymous, online survey 78. Hence, there is reason to believe delivering a direct questionnaire anonymously, online, may be suitable to examine base rates of risk factors for violent extremism in the general population.

However, online surveys too have a number of limitations. For example, the skewed attributes of online populations, sample representativeness (or a lack thereof), subjects’ lack of technological savviness, technological variations (desktop versus tablets versus mobile devices), unclear instructions, impersonality, privacy and security issues, and low response rates 75. Many of these limitations may be addressed by crowdsourcing samples via online panels.

Research has increasingly made use of online access panels such as Amazon’s Mechanical Turk (MTurk) 79. These panels are online platforms where users receive payment for their participation in research. Recently, a number of alternatives have emerged, one being Prolific. Prolific differs from MTurk in that it was created for researchers, in order to facilitate *academic* research. Therefore, it is explained to users that they will be participating in academic research upon registration. Research comparing MTurk, Prolific, and CrowdFlower (CF) finds the users of the latter two are more naïve and honest than MTurk users, a higher response rate yet higher rate of attention check failure in CF users, and that Prolific users produced data of comparable quality to MTurk’s, and better than CF’s 80.

Online panels are limited, however, in that they may be subject to a selection bias. More specifically, potential respondents are limited to those with Internet access, and those who register as panel users. This excludes a fair proportion of the general public and samples may therefore be limited in their representativeness. However, researchers who have predominantly relied on university student samples find online panels grant access to larger, more diverse samples than have traditionally been made available 80, and therefore, there is merit in crowdsourcing a sample through Prolific.

Indirect questioning techniques emerged in response to the problematic nature of directly measuring sensitive survey items 81. These include the Randomized Response Technique (RRT), the Nominative Technique, the Group‐answer Technique, the Diagonal Model, as well as the Unmatched Count Technique (UCT), also referred to as the Item Count Technique, or the List Item Technique which we employ here.

UCT necessitates two groups of respondents: a control condition and a UCT condition. Instead of directly self‐reporting the extent to which they agree with potentially sensitive attitudes, or engage in behavior that could be considered sensitive, respondents are asked how many items in a list of statements apply to them. Those in the control condition receive sets of nonsensitive items only. Those in the UCT condition receive the same set of items, with the addition of one item of interest. The difference between the mean number of responses endorsed by each group is inferred to be attributable to the proportion of respondents in the UCT condition who endorse the sensitive item.

UCT assumes that subjects do not fully trust their anonymity when self‐reporting sensitive items in direct surveys (and hence are subject to self‐reporting biases). By introducing an additional layer of anonymity, subjects may perceive their anonymity to be more robust, and hence report more accurate estimates of sensitive items. The UCT protocol has evidenced higher estimates of base rates of sensitive items than direct surveys 82, 83, 84, 85, 86, 87, 88, 89.

However, UCT is not without limitations. First, UCT requires relatively large sample sizes in order to be effective. Second, the protocol results in aggregate sample proportions rather than measures of the sensitive item for each respondent. This means that the data are not suitable for inferential testing such as regression modeling. This is a major limitation to consider, although Blair and Imai 90 and Glynn 81 describe strategies for conducting multivariate tests on responses derived from UCT questioning. Third, estimates are subject to sampling variance, particularly when utilizing multiple control items. Lastly, UCT can be subject to ceiling, and near‐ceiling effects (see Refs. 81 and 91 for a detailed discussion of these). However, as described, UCT has been shown to be effective in yielding higher estimates of base rates of phenomena that may be affected by reporting biases. Given the sensitivity and social undesirability of many risk factors of violent extremism (notably exposure), we employed UCT in our study.

## Method

This study employs two datasets: a preexisting dataset of 125 lone‐actor terrorists 13 and a dataset of survey respondents from a general population sample. First, we describe the lone‐actor terrorist data. Second, we detail the general population data collection and survey procedure.

### Lone‐Actor Terrorists

The defining criterion for identifying lone‐actor terrorist cases was whether subjects carried out or planned to carry out, alone, an attack in service of some form of ideology, for which they were convicted or died in the attempt. All individuals planned their attack in the United States, Europe, or Australia between 1990 and the end of 2015.

Information for each lone‐actor terrorist was identified based on a behavioral codebook of over 200 variables derived from the wider research literature which we extensively cite above 18. The initial lone‐actor terrorist study selected variables based on their presence in the research literature in order to estimate their actual presence in a sample of lone‐actor terrorists. It would therefore be incorrect to assume that each is a valid predictor of who engages in terrorism.

The data were compiled from open sources, including sworn affidavits, court reports, first‐hand accounts, and news reports obtained predominantly via LexisNexis searches. Additional sources such as biographies and scholarly articles were used where available and relevant. First, three independent coders coded the objective absence or presence of a behavioral indicator. Second, the three coders engaged in a two‐stage reconciliatory process. First, coder A compared observations of behaviors or experiences with coder B. Where differences were apparent, the original source documentation was checked for veracity by a senior researcher. Second, information identified by coders A and B was compared with coder C. Again, coding disparities were resolved by one of the principal researchers, who revisited the original sources and factored in the reliability of the documents when making decisions.

Where discrepancies occurred, decision‐making was guided by a “continuum of reliability,” where each source was plotted along a scale from “most reliable” to “least reliable.” Sources such as court transcripts and associated documents, for example, were considered the most reliable. Competency evaluations, sworn affidavits, and indictments were deemed reliable. Statements made by the offenders or affiliated groups were deemed somewhat reliable, as well as warrants and expert witness reports (which may be subject to unreliability and bias). Separately, media sources were also plotted along a reliability continuum where “least reliable” were sources such as personal opinion blogs and “most reliable” were nontabloid newspapers.

The mean age (at time of offence) of the lone‐actor terrorist sample was 33.56 years old (SD = 12.91). The sample was predominantly male (97.6%), with just 3 females (2.4%). At the time of their event, 48.8% (*n* = 61) of the sample were U.S. citizens.

### The General Population Sample

Subjects were recruited via an online panel, Prolific. In order to participate in the study, subjects were required to give informed consent. Participants were able to withdraw their consent at any point. In these instances, subject’s data were marked as “returned” and they were excluded from data collection. Their place in the study was reallocated to another potential subject, until the study quota was met. Seventy‐three participants “returned” their submissions. A further 40 participants failed to complete the study, and thus, their data were not retained.

Given the nature of the subject pool and to control for possible inattention, three attention checks were included 92. Some evidence suggests that excluding participants solely on the basis of a single attention check failure may result in bias 93. Hence, subjects who failed an attention check were escalated to a manual review of their data.

In review, researchers examined the length of time a subject spent completing the survey, the pattern of their responses (i.e., for scale items, was the same answer selected for every question) and whether they failed any other attention checks. Upon review of all of these factors, a decision was made about whether to reject or accept a submission. Based on these exclusion criteria, 42 submissions were rejected. The final sample size was 2,108. Participants ranged from 18 to 50 years of age, with a mean age of 30.06 years (SD = 8.43). The sample included 1158 (54.9%) females and 950 (45.1%) males. Of the sample, 52.1% were residing in the U.K., 28.4% in the United States, and 19.5% in Western Europe.

Participants were randomly assigned via a Qualtrics randomiser to one of three conditions: (1) direct survey, (2) UCT control, or (3) UCT treatment. Wimbush and Dalton 82 suggest that with sufficient sample size and random assignment, moderate differences in sample size should not impact upon outcomes. There were no significant differences in the terms of the demographics of the groups (see Table 1). Either analysis of variance or chi‐square tests assessed group differences.

**Table 1:** Socio‐demographic descriptive statistics for all conditions.

	Direct Survey (*n* = 706)	UCT Control (*n* = 703)	UCT Treatment (*n* = 699)	*p* Value
Age (in years)	29.9	29.9	30.3	0.54
Sex				0.60
Male	46.6%	44.4%	44.2%	
Female	53.4%	55.6%	55.8%	
Socioeconomic status*	5.1	5.1	5.3	0.18
Current place of residence				0.62
U.K.	50.7%	50.9%	54.4%	
U.S.A.	30.2%	27.6%	27.2%	
Western Europe	19.1%	21.5%	18.4%	
Highest education level				0.34
No formal qualifications	1.6%	1.7%	1.7%	
Secondary school/GCSE	15.4%	16.9%	18.1%	
College/A Levels	26.9%	30.9%	28.3%	
Undergraduate degree	35.6%	33.9%	31.0%	
Graduate degree	18.1%	13.8%	17.7%	
Doctorate degree	2.3%	2.8%	3.0%	
Prefer not to say	0.1%	0.0%	0.0%	
Employment status				0.70
Full‐time	44.1%	44.4%	47.9%	
Part‐time	20.8%	19.8%	18.9%	
Due to start a new job	2.1%	3.0%	2.9%	
Unemployed/job‐seeking	14.0%	14.5%	13.4%	
Not in paid work	9.5%	8.8%	9.9%	
Other	9.5%	9.5%	7.0%	
Prefer not to say	0.0%	0.0%	0.0%	
Marital status				0.20
Single	35.3%	36.1%	32.6%	
In a relationship	33.1%	38.1%	36.3%	
Married	27.8%	22.2%	27.8%	
Separated	1.3%	0.3%	1.0%	
Divorced	1.3%	1.7%	1.4%	
Widowed	0.3%	0.4%	0.1%	
Other	1.0%	1.1%	0.7%	
Prefer not to say	0.0%	0.0%	0.0%	

* Measured using the Macarthur scale of subjective social status (Adler & Stewart, 2007) 94.

### Measures

All variables from the lone‐actor terrorist codebook that did not refer directly to committing terrorist offences (e.g., preparing explosive devices for an attack) were translated to survey items. Exceptions were items, which called for temporal sequencing (as this was not within the scope of the present study) or in‐depth elaborations (i.e., details of multiple prior arrests). Hence, the survey is collated from all observable behavioral indicators from the lone‐actor codebook. The survey questionnaire is hosted on the Open Science Framework (OSF) here (https://osf.io/gjx4q/).

Participants first answered questions relating to their life experiences, attitudes, and any behaviors of interest that they may have witnessed. This is part 1 of the survey. Situational variables were coded as present in the lone‐actor terrorist sample if they occurred in the build‐up to an attack. In the general population sample, participants were asked to report how many situational stressors occurred during the past year. This was in order to capture experiences of acute stress, rather than the occurrence of stressors over a lifetime. Attitudinal items and psychological constructs reported in the lone‐actor terrorist codebook as absent/present were not self‐reported here, given the inherent biases of doing so. Instead, these items were measured with preexisting scale items.

Self‐control was measured with five statements drawn from items used and developed by Grasmick, Tittle, Bursik, and Arneklev 95 (“When I am angry, other people better stay away from me,” “I lose my temper pretty easily,” “I often act on the spur of the moment without stopping to think,” “I often get into trouble because I act without thinking,” “I never think about what will happen to me in the future”). Thrill‐seeking was measured with three items (“I often do things without thinking of the consequences,” “Sometimes I will take a risk just for the fun of it,” “I sometimes find it exciting to do things that might be dangerous”) 96. Overconfidence/grandiosity was measured with two items derived from Peters, Joseph, Day, and Garety 97’s 21‐item Delusions Inventory (“I am destined to be someone very important,” “I am very special”). All scale items were scored along a seven‐point Likert scale ranging from “strongly agree” to “strongly disagree.”

Thrill‐seeking, self‐control, and overconfidence/grandiosity were dichotomized post data collection in order to facilitate comparison. First, Cronbach’s alpha was calculated for each scale: thrill‐seeking (α = 0.76), self‐control (α = 0.81), overconfidence/grandiosity (α = 0.84). Second, a mean score was calculated for each participant. Lastly, scores were dichotomized by converting scores of ≤3 (i.e., “strongly agree,” “somewhat agree,” “sort of agree” on the Likert scale) to present, and all other values to absent. This reflects the coding of the lone‐actor terrorist data that were deemed to demonstrate evidence of the trait (present) or not (absent).

Three items inspired by the VERA 98, conceptualized as protective factors, were included. Protective factors are sometimes included in the assessment of violent risk as factors which may mitigate the likelihood of future violence. These were “community support for nonviolence,” “family support for nonviolence,” and “rejection of violence to obtain goals.” These items were translated for use with a general population sample. For example, the item “community support for nonviolence” specifically relates to violent extremism in the VERA. Rather, we asked “does your community disapprove of others committing acts of violence?” This is an approximation of a protective factor inspired by the VERA but does not measure precisely the same information. The first two items were measured as dichotomous yes/no items to reflect the coding of the VERA. The latter, an attitudinal item, was recorded along a 7‐point Likert scale from “strongly agree” to “strongly disagree” (“It is OK to use violence to achieve my goals”). As above, this item was dichotomized by converting scores of ≤3 (i.e., “strongly agree,” “somewhat agree,” “sort of agree” on the Likert scale) to present, and all other values to absent.

#### Direct Questioning

All items from the lone‐actor terrorist codebook deemed sensitive were presented as a traditional self‐report survey (see Table 3). Indicators relating to exposure were translated directly from the lone‐actor terrorist codebook. Additionally, the codebook includes items that measure leakage. Leakage refers to the extent to which a person signals, directly or indirectly, their intent to engage in violence. These items were translated to measure the extent to which the general population may have witnessed leakage behaviors. For example, “Have you ever witnessed someone make verbal statements in support of a violent ideology?” was translated from the codebook item “Did the individual make verbal statements in the build‐up to their attack?” These items serve as a further proxy for exposure to extremism in the general population, however, may also serve as a crude marker for the prevalence of extremism in the general population.

#### Indirect Questioning (UCT)

Participants were either in the control group, or the UCT group. As previously outlined, participants were presented with a series of lists of items and selected the number of statements they endorsed from a multiple‐choice list, ranging from numbers 0 to 5 in the control condition, and 0 to 6 in the UCT condition. It was therefore not possible for participants to signal, or researchers to know, which statements may be true for respondents. As participants were assigned to each condition randomly, there should be no significant group differences, as can be seen in Table 1. Therefore, any difference in the mean number of statements endorsed can be attributed to endorsement of the sensitive item in the treatment condition. Wimbush and Dalton 82 suggest that the minimum group size for UCT should be 40 – 50 subjects. The present study utilized samples of approximately 700 (control condition = 703, UCT condition = 699). A control item was included to act as a measure of UCT’s effectiveness (“I have read (online or offline) material from any political group.”). See the supplementary material for a more detailed explanation of UCT and how to calculate base rates estimates.

### Procedure

Prolific allows researchers to constrain the potential subject pool by a number of prescreening questions. These are items which potential respondents self‐report upon registration to Prolific. Of the approximately 70,000 potential subjects, we limited the sample to those aged 18–50 years old, who currently resided in the U.K., the United States, or Western Europe. This identified a potential pool of approximately 27,000 participants, from which we recruited.

The survey was administered online, hosted by Qualtrics and delivered exclusively via Prolific. We collected the prescreening data for a number of demographic items. These were current place of residence, sex, highest education level, marital status, socioeconomic status, employment status (see Table 1), and whether or not they had children. Subjects were paid at a rate of approximately £5.00/h for participating in the survey, estimated to take 15–20 min after piloting. There were no missing values.

## Results

A criterion for measuring the effectiveness of UCT is whether the protocol elicited higher base rate estimates of sensitive items than the direct survey 82, 99. In the present study, this was largely not the case. In light of this, and after further investigation, we utilized the results from the direct survey in a series of comparisons with the lone‐actor terrorist population. The following section first presents a comparison of the base rates obtained from the two survey designs. Second, we compare the prevalence of risk factors for violent extremism between the lone‐actor terrorist and the general population sample. For a table summarizing all base rate estimates, see Appendix [Link] in the supplementary materials.

### UCT Protocol

Given that the UCT condition received sets consisting of six items, it follows that the mean number of statements endorsed should be higher than the control condition (who received sets of five items). This was not the case for 17 of the 25 risk factors. To investigate further, we conducted a multivariate analysis of variance. Box’s Test was significant and four dependent variables violated assumptions of equality of variance. Multivariate analysis of variance is fairly robust against violations of these assumptions, given large and relatively equal sample sizes, as we have here, hence we proceeded. The analysis was significant for condition (*F* (25, 1376) = 7.17, *p* < 0.000; Wilk’s A = 0.115, partial η^2^ = 0.12). After correcting for multiple comparisons (*p* < 0.002), we found two significant differences, as can be seen in Table 2.

**Table 2:** Multivariate analysis of variance of the 25 sensitive survey items obtained via indirect questioning for the control and UCT conditions. Alpha adjusted for multiple comparisons (*p* < 0.002).

Item	df	df Error	*F* Statistic	partial η^2^	Condition	Mean	Mean Diff	Std. Error	Estimated Base Rate
Engaged with the materials of any political group (control)	1	1400	28.17	0.020	Control UCT	1.81 2.09	0.274	0.052	27.4%***
Virtual interactions with extremists online	1	1400	67.18	0.046	Control UCT	1.15 0.75	−0.403	0.049	−40.3%***

Item	Condition	Mean	Mean Diff	Std. Error	Estimated Base Rate
Required support as a child	Control UCT	3.18 3.00	−0.176	0.061	17.6%
Expressed a desire to hurt others	Control UCT	2.47 2.62	0.150	0.052	15.0%
Engaged with materials about lone‐actor terrorists	Control UCT	1.20 1.31	0.104	0.045	10.4%
History of substance abuse	Control UCT	1.62 1.72	0.102	0.056	10.2%
Perpetrated domestic abuse	Control UCT	2.68 2.75	0.068	0.062	6.8%
Family members made verbal statements in support of violence	Control UCT	1.52 1.58	0.060	0.054	6.0%
Close associates involved in criminality or extremism	Control UCT	1.57 1.61	0.033	0.054	3.3%
Participated in high‐risk activism	Control UCT	1.82 1.83	0.006	0.054	0.6%
Joined a wider extremist group	Control UCT	2.39 2.17	−0.220	0.069	−22.0%
Rejected from a political group	Control UCT	2.28 2.12	−0.163	0.058	−16.3%
Previous criminal convictions	Control UCT	2.59 2.53	−0.067	0.052	−6.7%
Violent as a child	Control UCT	2.14 2.11	−0.032	0.061	−3.2%
Extreme views	Control UCT	1,96 1.94	−0.022	0.059	
Previously imprisoned	Control UCT	2.43 2.38	−0.055	0.053	−5.5%
Searched online for extremist materials	Control UCT	1.90 1.86	−0.033	0.062	−3.3%
Committed an act of violence as an adult	Control UCT	1.26 1.23	−0.044	0.050	−4.4%
Spouse involved in extreme political movement	Control UCT	1.58 1.51	−0.070	0.056	−7.0%
Face‐to‐face interactions with members of an extremist group	Control UCT	2.17 2.17	−0.003	0.048	−0.3%
Access to a stockpile of weapons	Control UCT	2.41 2.31	−0.095	0.064	−9.5%
Tried to recruit others to form an extremist group	Control UCT	1.61 1.59	−0.021	0.060	−2.1%
Engaged with the propaganda of an extremist group	Control UCT	1.49 1.45	−0.046	0.052	−4.6%
Engaged with propaganda of lone‐actor terrorists	Control UCT	2.04 1.98	−0.061	0.055	−6.1%
Arrested as a juvenile	Control UCT	2.14 2.11	−0.028	0.052	−2.8%

*** *p* < 0.002.

First, 27.4% of the UCT group endorsed the control item, *engaged with materials from any political group*. In the remaining instance, we found a negative estimate, that is, a deflation effect. The estimated prevalence of the item *engaged in virtual interactions with extremists online* was −40.30%. However, it is important to note the small effect sizes when considering these findings.

Subsequently, we compared the results of the UCT protocol with the direct questioning protocol (see Table 3). *Z*‐tests were used to compare the two proportions. Only positive values were compared, as a negative proportion here is illogical.

**Table 3:** Estimates of the base rates of sensitive items from the UCT and direct survey protocol.

Items	UCT Condition (*n* = 699)	Direct Survey (*n* = 706)	Std Error	Lower Bound 95% CI	Upper Bound 95% CI
Engaged with the materials of any political group (control)	27.4%	56.1%***	0.026	0.235	0.338
Engaged with propaganda about other lone‐actor terrorists	10.4%	18.7%***	0.019	0.046	0.120
Perpetrated domestic abuse	6.8%	10.1%*	0.015	0.004	0.062
Family made verbal statements in support of political violence	6.0%	4.4%			
History of substance abuse	10.2%	9.5%			
Expressed a desire to hurt others	15.0%	12.8%			
Participated in high‐risk activism on behalf of a group	0.6%	0.1%			
Close associates involved in criminality or extremism	3.3%	1.7%			
Previous criminal convictions	−6.7%	2.6%			
Violent as a child/adolescent	−3.2%	5.1%			
Extremist views	−2.2%	4.3%			
Previously imprisoned	−5.5%	1.3%			
Required additional support as a child	−17.6%	8.1%			
Searched online for extremist materials	−3.3%	7.1%			
Committed an act of violence as an adult	−4.4%	6.8%			
Spouse involved in extreme political movement	−7.0%	0.9%			
Face‐to‐face interactions with members of an extremist group	−0.3%	7.2%			
Access to a stockpile of weapons	−9.5%	3.3%			
Virtual interactions with extremists online	−40.3%	10.9%			
Joined a wider extremist group	−22.0%	0.1%			
Rejected from a political group	−16.3%	0.6%			
Attempted to recruit others to form an extremist group	−2.1%	0.1%			
Engaged with the propaganda of an extremist group	−4.6%	19.6%			
Engaged with propaganda by lone‐actor terrorists	−6.1%	11.9%			
Arrested as a child/adolescent	−2.8%	5.0%			

*** *p* < 0.000, *p < 0.05.

The results suggest that the UCT protocol did not elicit higher base rates than the direct survey protocol. Therefore, for the survey items deemed sensitive, the base rate estimates obtained from the direct survey protocol were employed for comparison with the lone‐actor terrorist sample.

### Comparing Lone‐Actor Terrorists With a Sample from the General Population

The following section compares the prevalence of a number of risk factors of violent extremism conceptualized as relating to propensity, situation, and exposure, between lone‐actor terrorists and a sample from the general population. We use chi‐square and Fisher’s exact tests where appropriate. As outlined above, all nonsensitive items were asked of the full sample (*n* = 2108). Estimates of the base rates of items deemed sensitive are reported from the results of the direct survey condition, only (*n* = 706).

#### Propensity

**Figure 1:**
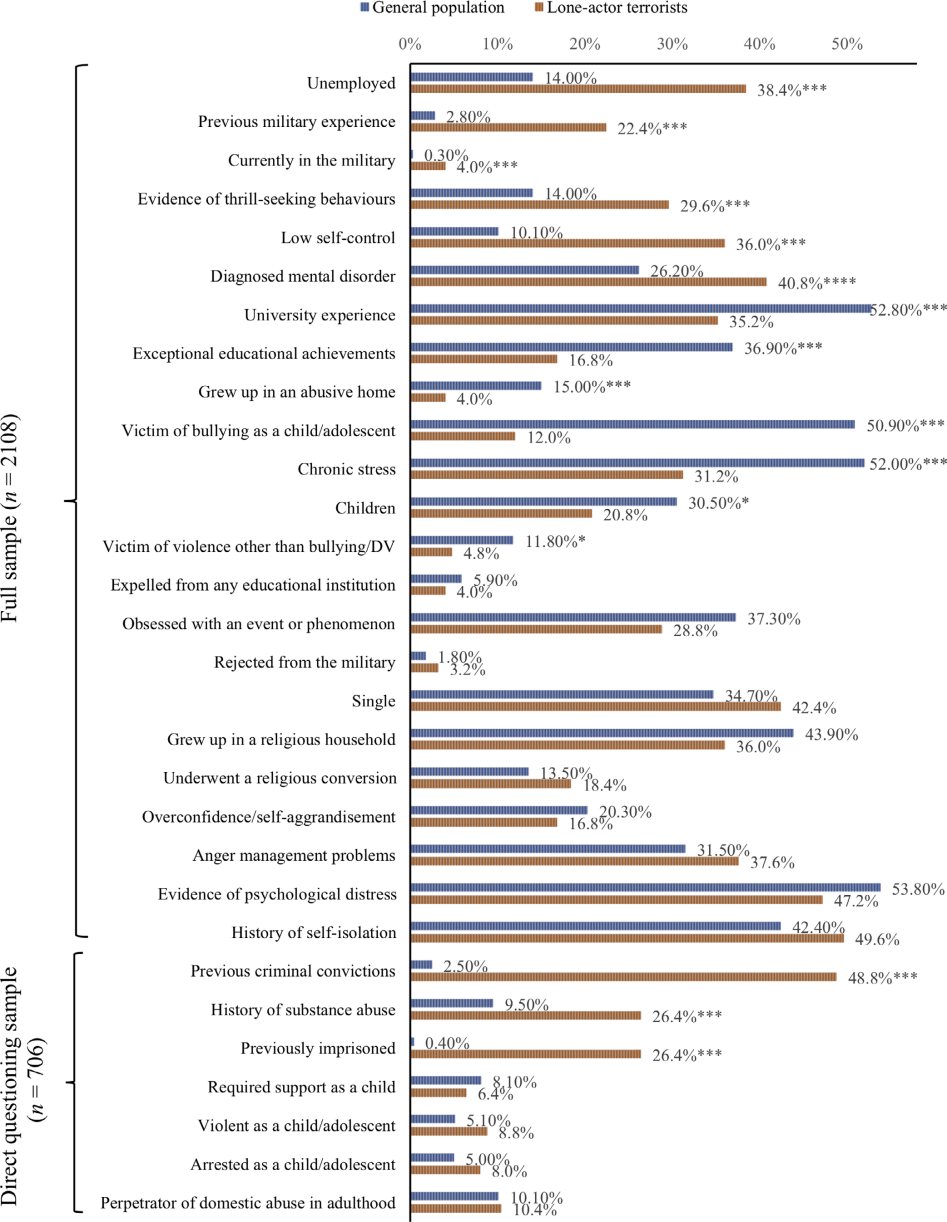
A comparison of lone‐actor terrorists with a sample from the general population across propensity indicators (***p < 0.000, *p < 0.05). [Color figure can be viewed at wileyonlinelibrary.com]

We find a number of significant differences (Figure 1). Table S2 in the supplementary materials provides further detail including standard error statistics and confidence intervals.

Lone‐actor terrorists were significantly more likely to have previous criminal convictions, previously been in prison, a history of substance abuse, previous military experience, or be in the military (at the time of their terrorist event), demonstrate evidence of thrill‐seeking, low self‐control, diagnosed mental disorder, and be unemployed. The general population sample was more likely to have children, university experience, exceptional educational achievements, experienced bullying as a child/adolescent, chronic stress, or experienced violence other than bullying or domestic violence.

#### Situation

**Figure 2:**
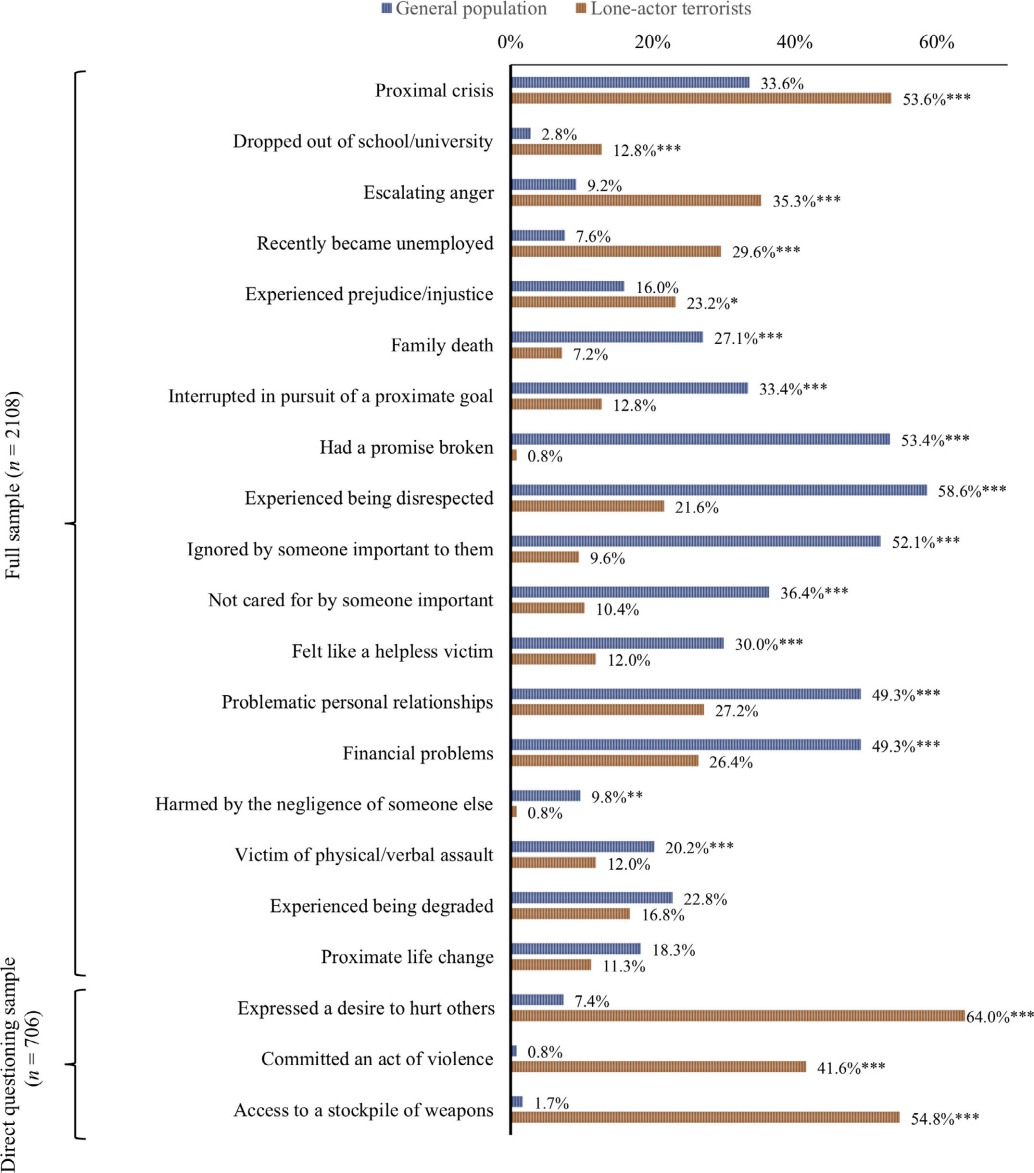
A comparison of lone‐actor terrorists with a sample from the general population across situation indicators (***p < 0.000, **p < 0.01, *p < 0.05). [Color figure can be viewed at wileyonlinelibrary.com]

Figure 2 summarizes comparisons between the lone‐actor terrorist and the general population sample across indicators conceptualized as situational factors. Table S3 in the supplementary materials provides further detail.

Lone‐actor terrorists were significantly more likely to have recently been made unemployed, experienced proximal crisis, prejudice or injustice, escalating anger, and to have dropped out of school/university. The general population sample were more likely to have experienced a death in the family, been interrupted in pursuit of a proximate goal, had a promise broken, been disrespected, ignored by someone important to them, felt like a helpless victim, problematic personal relationships, financial problems, harm due to the negligence of someone else, and been the victim of physical or verbal assault. The general population sample also reported three items inspired by protective factors included in the VERA. First, 85.1% of the general population sample reported that their community disapproves of violence. Second, 89.0% reported that their family disapproves of violence. Lastly, 12.8% reported attitudes in support of violence.

#### Exposure

Table 4 displays the results of a series of exposure‐related items that asked the general population sample to what extent they had witnessed certain behaviors.

**Table 4:** The prevalence of witnessed or observed behaviors in a general population sample.

Exposure Indicators	General Population (*n* = 2108)
Witnessed someone produce letters/public statements	13.4%
Witnessed someone make verbal statements to a wider audience	33.9%
Witnessed a direct threat of extremist violence	7.8%
Aware of someone else's grievances	23.3%
Aware of someone else's extremist ideology	22.6%
If yes, what was their ideology?	
Right wing	5.7%
Nationalist	4.5%
Religious	3.7%
Left wing	3.6%
Single issue	3.6%
Other	1.7%
Did they commit an act of extremist violence?	3.7%
If yes, did their religious beliefs intensify in the buildup?	0.5%
If yes, did their ideological beliefs intensify in the buildup?	1.9%
In the buildup, did they change religions?	0.4%

	Direct Sample (*n* = 706)	
	Lifetime Prevalence	In the Last Year
Have you searched for extremist content online?	6.8%	3.7%
Have you ever held extremist beliefs	4.2%	2.3%

**Figure 3:**
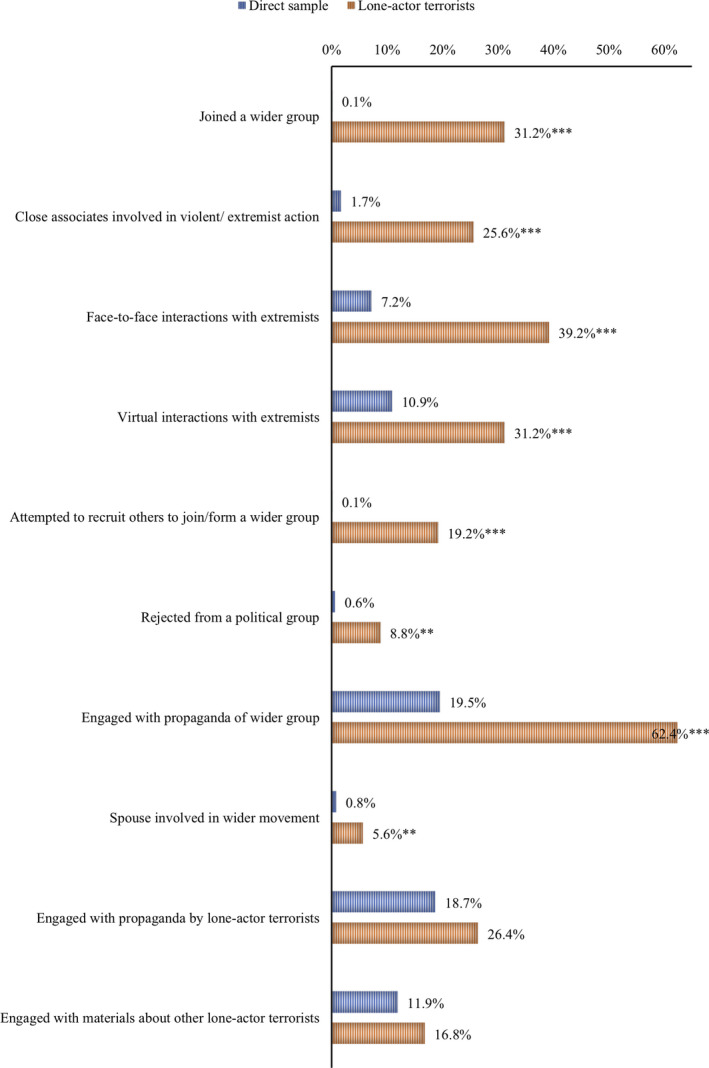
A comparison of lone‐actor terrorists with a sample from the general population (*n* = 706) across exposure indicators (***p < 0.000, *p < 0.05). [Color figure can be viewed at wileyonlinelibrary.com]

Figure 3 presents a comparison of further exposure‐related indicators with the lone‐actor terrorist sample. Table S4 in the supplementary materials provides further detail.

Lone‐actor terrorists were significantly more likely to demonstrate evidence of all but two exposure indicators; engaged with propaganda by lone‐actor terrorists (i.e., manifestos) and engaged with materials about lone‐actor terrorists. The former was found to be significant (*p* < 0.05), however, the CI included 0 and so we reject this result.

Lastly, we present a comparison of the mean number of propensity, situational, and exposure indicators between the two samples. Whilst multivariate analysis of variance is fairly robust against violations of its assumptions, as previously stated this is largely only the case when considering large and equal sample sizes. This was not the case here, and so a series of independent sample *t*‐tests were conducted (alpha adjusted for multiple comparisons).

Table 5 displays a comparison of the mean number of propensity and situational indicators (sensitive and nonsensitive). First, there was no significant difference between the mean number of nonsensitive propensity indicators experienced by the two groups. The general population sample experienced significantly more nonsensitive situational indicators than lone‐actor terrorists, *t*(155.03) = 8.245, *p* < 0.000, *d* = 0.76). Second, lone‐actor terrorists experienced significantly more sensitive propensity *t*(143.86) = −7.908, *p* < 0.000, *d* = −0.77), situation *t*(129.37) = −18.290, *p* < 0.000, *d* = −1.78), and exposure *t*(138.75) = −9.591, *p* < 0.000, *d* = −0.93) indicators than those in the general population sample.

**Table 5:** A comparison of the mean number of propensity, situation, and exposure indicators (sensitive and nonsensitive) in lone‐actor terrorists and a general population sample.

	General Population (*n* = 2108)	SD	Lone Actor (*n* = 125)	SD	Mean Difference	Std Error Difference	Lower Bound 95% CI	Upper Bound 95% CI
Propensity (non‐sensitive)	5.63	2.64	5.66	2.89	0.12			
Situational (non‐sensitive)	5.30	3.75	3.23	2.65	2.07***	0.2506	1.571	2.561
Propensity (sensitive)	0.41	0.85	1.35	1.28	0.94***	0.119	−1.173	−0.704
Situational (sensitive)	0.10	0.31	1.60	0.91	1.5***	0.082	−1.662	−1.337
Exposure (sensitive)	0.72	1.27	2.66	2.21	1.95***	0.203	−2.347	−1.544

*** *p* < 0.000.

## Discussion

This study presents the first estimates of base rates of common risk factors/indicators associated with violent extremism in a general population. To the best of our knowledge, it is the first study entirely dedicated to establishing the base rates of risk factors for any type of violence. We compared survey data collection methodologies and found that direct questioning was most appropriate (although this is not without limitations). First, we consider the implications of base rates for the risk and threat assessment of violent extremists. Second, we discuss how lone‐actor terrorists differ from our general population sample, and what this may mean for managing risk in these populations. Third, we consider the implications of our findings for survey methods in terrorism research.

### Base Rates and Risk Assessment

The development of base rates of risk and protective factors will impact upon different forms of risk and threat assessment differently. For example, actuarial methods focused upon risk prediction fundamentally depend upon the development of empirically established risk factors. Developing base rates and predictors of various risk specifications are important steps, alongside many others, to such an establishment. For the assessment and management of violent extremism, actuarial methods may have the greatest utility for triage and case prioritization processes when volume is high, but resources are finite. Actuarial approaches are not suitable for all stages of the risk assessment and management process 100, 101, 102, 103.

The limitations of actuarial approaches include generalizability beyond the samples used in development of a tool, the challenge of applying statistical knowledge to a clinical setting, the propensity of actuarial methods to exclude potentially important risk factors, rigidity of actuarial models and their lack of space for change, and failure to address violence prevention and risk management. In addition, the actuarial method has the potential to disregard the different dynamics of risk, including the nature, severity, imminence, duration, and frequency of future violence 104. Further, Hart et al. 105 argued that although actuarial methods are reasonably reliable for group estimations of risk, they are not reliable for estimations of an individual’s risk of future violence.

The utility of base rates is different for those risk assessment processes more reliant on human judgement and where the goal is risk prevention. Base rates can assist clinical unstructured approaches, which likely underestimate the frequency of exposure‐related behaviors or overestimate the frequency of other suggested causes based on the practitioner’s memory of previous empirical findings, and perhaps intuition 106.

Structured Professional Judgement (SPJ) approaches encourage practitioners to review all available clinical data to identify any potential risk factors, which are found in a structured manual based on empirical evidence. Based on these factors, a final structured risk judgement is made, which indicates the risk of violence 107. Unlike actuarial methods, SPJ does not include fixed guidelines on how to calculate level of risk; instead, SPJ tools are structured to guide the decision‐making process of practitioners. Tools in this category include a list of risk factors, all of which have been empirically supported, with guidelines on how these risk factors are scored and on how to reach a final judgement of different gradations of risk 108. SPJs therefore require the inclusion of valid factors and indicators in any tool to guide the professional’s judgment. The development of base rates is one of many important steps toward this goal.

It is important to note that for both our scientific understanding of causation and for practical and more in‐depth SPJs of potential violent extremist risk, it is insufficient to only examine the presence of indicators. Instead, we need to additionally understand relevance. Just because a factor such as problematic personal relationships is more likely in the general population, does not mean it should not be considered when judging extremist risk. It might be highly relevant to understanding particular cases. Other types of research design may be more important for the issue of relevance.

Indeed, the general population sample was significantly more likely to experience a range of, and in fact more of, a number of situational stressors. This is not to say that acute strain is unimportant in understanding trajectories to violent extremism. In fact, a number of studies have demonstrated the role of acute and general strain in various types of targeted violence 19, 60, 108. Instead, these findings highlight the problem of specificity, that is, being able to correctly differentiate between true positives (i.e., terrorists) and true negatives (i.e., nonterrorists), who may share some characteristics 6. A significant proportion of the general population experience a number of strains and stressors (in the present instance, more so than our lone‐actor terrorist sample), yet do not go on to commit terrorism. However, acute strain may act as a catalyst, or tipping point, alongside the co‐occurrence of individual‐level susceptibilities, further situational factors, and varying degrees of exposure.

One step in the SPJ process is the generation of a statement of understanding about the case (e.g., the formulation). Evaluations of formulations are beginning to grow “based on the premise that the quality of case formulations may impact on outcomes” (109, p. 32). Bucci et al.’s 110 systematic review found eight separate quality assessment measures of case formulations. One consistent feature of these assessment measures concerns external coherence (e.g., the degree to which it is consistent with empirically supported theory). The development of base rates is key to this particularly when we consider issues concerned with equifinality and multifinality.

Lastly, consideration of protective factors is a key component of the SPJ approach. Such factors are not currently *explicitly* mentioned in most violent extremist risk assessment tools, with the exception of the VERA and its later iterations. Protective factors/circumstances are considered in the Extremism Risk Guidelines for each of the 22 factors identified (but not as distinct additional factors as per the VERA). Our findings provide further empirical evidence for the consideration of protective factors in violent extremist risk assessment, thereby supporting SPJ approaches which have generally shown utility in the assessment for general violence 111.

### Risk Factors in Lone‐Actor Terrorism

There were noticeable significant differences between our existing lone‐actor terrorist sample and the general population sample. In terms of propensity‐related indicators, lone‐actor terrorists were significantly more likely to display indicators inferred to be proxy measures for a cognitive vulnerability 13, 19. For example, lone‐actor terrorists were significantly more likely to have a diagnosed mental disorder than the general population sample, thus replicating Corner et al. 57. Lone‐actor terrorists were also significantly more likely to display low self‐control and thrill‐seeking behaviors, as found by Nussio 66, as well as proxy indicators of a crime‐ and/or violence‐supportive morality, such as having a history of substance abuse, having a criminal conviction and/or experiencing imprisonment, violence unrelated to terrorism, and escalating levels of anger. These results mirror previous conceptualizations of violent extremism as resulting from a dynamic interaction among specific individual‐level vulnerabilities (i.e., cognitive and/or moral susceptibilities), various situational factors, and differential exposures to terrorism‐supportive settings and/or moral norms, 13, 18, 19, 20. Unsurprisingly, lone‐actor terrorists were significantly more likely to express a desire to hurt others, and to have had access to a stockpile of weaponry.

The general population were significantly more likely to experience a range of distal stressors such as growing up in an abusive home, being a victim of bullying, and experiencing chronic stress. Despite the greater levels of distal stressors within this sample, they were also more likely to display factors often considered to be protective against criminal engagement, such as university experience, being employed, exceptional educational achievement, and having children. Similarly, in the general population sample, we observed high prevalence estimates of three protective factors inspired by the VERA. We do not have data relating to these measures for the lone‐actor terrorist sample; however, this would be a useful avenue for future research, and again points to the importance of considering protective factors in the assessment of violence risk.

Again, unsurprisingly, lone‐actor terrorists were significantly more likely to demonstrate a range of indicators of exposure to violent extremism. However, the value in the results here is in demonstrating the prevalence at which these occur within the general population. A large minority of the general population sample were aware of someone in their network’s adoption of an extremist ideology (22.6%) and engaged with extremist propaganda (19.5%). Smaller numbers witnessed direct threats of extremist violence (7.8%), or directly interacted with extremists both offline (7.3%) and online (10.9%), or associated with individuals involved in violent extremist action (1.7%). Some of these results may be larger than one might expect and reiterate the importance of considering *configurations* of risk factors in trajectories to violent extremism 19.

As previous research demonstrates, there is rarely a single factor driving violent extremism. It is usually a crystallization of multiple push and pull factors 112. Whilst many indicators were more likely among the general population sample, or demonstrated no significant difference between the samples, on average, lone‐actor terrorists were more likely to experience a greater number of indicators inferred to relate to a cognitive vulnerability, a crime‐ and/or violence‐supportive morality, and exposure to extremism. However, no single factor can easily discriminate between the samples.

### Survey Methods in Terrorism Research

We undertook a test of different survey methods to capture base rates of risk factors for a number of reasons. First, to the best of our knowledge, no previous studies have attempted to estimate base rates of violent extremism risk factors/indicators in a general population. Therefore, we have little to draw from to evaluate the validity of any estimates we obtained. That being said, base rates of more general indicators such as mental illness are available and generally well‐established. For example, the lifetime prevalence of any mental disorder in a general population is reported as 25.0% (CI 95 24.2–25.8), which mirrors what we established here (26.2%) 113. However, estimates of how often the general population engage with extremist propaganda or interact face‐to‐face with extremists were not readily available.

Second, establishing the base rates of sensitive items is challenging. As previously described, direct surveys may be subject to a number of biases, however, indirect surveys may similarly be affected by sampling variance, ceiling effects, and deflation (as observed here). Third, given the relative recency of crowdsourcing samples, particularly in terrorism studies, it is necessary to test the functionality of different survey methods in potentially novel populations. Indirect questioning emerged predominantly from reporting biases observed in traditional research settings (i.e., face‐to‐face, or interviewer present). Conducting research online with panels may have important differences, as the present results suggest. Hence, this was a necessary first step, and further research to test (by replication) these findings is necessary (see the supplementary materials for a more in‐depth discussion of the results of UCT).

### Limitations

The present study is not without limitations. First, we consider the constraints of the lone‐actor terrorist data. The data were drawn from open‐source material. Open‐source data have been criticized for having the potential to be unreliable, subject to bias, and incomplete 114. However, open‐source data have been the source of a range of important findings. Robust data collection methodologies and provisions to ensure intercoder reliability can mediate many of these concerns, as in the present study.

Second, open‐source data are characterized by high levels of missing data and biases with regard to the nature of what is missing (the availability bias). Researchers should be transparent about the assumptions made about missing data and the effects of missing values on any recommendations (see Ref. 115). Given the nature of the data, there is likely to be some underreporting of certain types of indicators. For instance, the indicator *had a promise broken* is reported infrequently in the lone‐actor data, but is much more prevalent within the general population sample. Rather than suggesting that lone‐actor terrorists do not experience this indicator, this may be subject to the availability bias.

Third, it is important to consider the treatment of missing data. When relying on open‐source reporting, it is sometimes difficult to differentiate between missing data and data that should be coded as “no” or as “not present.” The authors of these sources, such as journalists, are unlikely to report at great length the absence of potentially infinite indicators that may be of interest to researchers 49. However, in previous research on attempted assassinations of public figures, fatal school shootings, targeted violence affecting higher education institutions, and terrorism, researchers have employed similar strategies 18, 116, 117.

Limitations of the survey and survey data should also be considered. First, the sample was not representative. At the time of conducting this research, Prolific began testing a beta version of a functionality that would allow researchers to generate a representative sample in the U.K. and the United States. This may be a promising development for future research, particularly in any attempt to replicate the present findings. Second, whilst we suggest that in the present case, direct questioning elicited the best results, we do not suggest that these are not also subject to self‐reporting biases. Gomes et al. 74 recently conducted a systematic review of measurement biases in self‐reports of offending behavior and demonstrated the range of potential biases that may occur under varying conditions. No design will be absent of these, however, some may be more suited to certain study designs than others, as in the present study.

Third, Prolific users, whilst more naïve than MTurk users, are not a naïve sample population. It is important to consider the implications this may have for any applications of our findings. However, traditional survey samples such as student populations are equally, perhaps more so, experienced research participants, and so, Prolific may provide researchers with access to a relatively novel population. Fourth, the present UCT design was drawn from previous studies that successfully elicited higher base rate estimates of sensitive items. However, some have suggested strategies to design against potential negative effects, some of which we may have succumbed to here. For instance, Glynn 81 recommends using negative within‐list correlations to reduce variance and bias due to ceiling effects. This too may be a promising avenue for future research to consider.

Fifth, all items were drawn directly from the lone‐actor terrorist codebook in order to facilitate direct comparison. However, some questions may have been more difficult to comprehend or more open to interpretation given that they were not designed as survey items. As a first step toward developing base rates, we felt this a necessary limitation to accept. However, future research should aim to refine these items when seeking to replicate these findings. The Base Rate Study survey is hosted on the OSF and is freely available to encourage collaborative refining.

Lastly, in comparing the general population and lone‐actor terrorist dataset, the different data collection methodologies should be considered. The lone‐actor terrorist dataset was collated by researchers following a rigorous and robust open‐source data collection methodology. The general population sample, however, answered questions directly relating to these experiences. That is, we compare information drawn from secondary sources to that of self‐evaluation, where both do not refer to the same subject. Related to the previously mentioned points, the fact that the latter demonstrated significantly more situational stressors may be due in part to those experiences being more accessible when self‐reporting this information, and less accessible, because less reported on, when relying on third‐party sources of data. Future research should consider comparing base rates identified through direct questioning within a general population and a terrorist sample. Having said this, given the difficulties engaging a sufficiently high number of terrorists in a single research design, we believe that our approach still provides valuable information.

## Conclusion

The base rate study is the first step toward establishing general population estimates of risk factors and indicators associated with violent extremism. It is necessary to seek to replicate these findings in order to provide robust estimates for use in evaluating and designing risk assessment tools. More generally, the present study provides evidence for crowdsourcing samples, particularly in terrorism studies. Whilst Prolific is often employed in social science research, its use in terrorism research is limited. We suggest it may be of substantial benefit for future research considering its ease of use, accessibility, and wide reach. Considering future directions, whilst the present study was descriptive and aimed to provide the first estimation of the above‐mentioned base rates, our results suggest the basis for testable hypotheses for factors which may differentiate between violent extremists and the general population. Furthermore, our findings suggest that some factors may be associated with exposure to extremism, and hence, future work will examine predictors of who in our general population sample self‐reported engaging in behaviors associated with violent extremism (e.g., looking at propaganda, engaging with extremists, holding extremist views). Finally, we further suggest examining how these individual factors/indicators may interrelate dynamically, where we consider observable patterns of indicators that may crystallize in time and space, in both trajectories to violent extremism, and within a general population sample.

## Supporting information

Appendix S1. Supplemental Information.
