# Supplementary material for: The Base Rate Study: Developing Base Rates for Risk Factors and Indicators for Engagement in Violent Extremism
Source: J Forensic Sci. 2020 Jan 30;65(3):865–81. doi: 10.1111/1556-4029.14282 (PMC7318282; doi:10.1111/1556-4029.14282)
Supplement: Supplementary file 1 — Appendix S1. Supplemental Information. [file JFO-65-865-s001.docx]

**Supplemental Material**

*How to Calculate Base Rates*

Any difference in the mean number of statements endorsed can be attributed to endorsement of the sensitive item in the treatment condition. For example, if the mean number of responses for set 1, in the control condition, was 2, and the mean number of responses for set 1, in the UCT condition, was 2.7, the mean difference, 0.7, could be interpreted as the proportion of subjects endorsing the additional item in the UCT condition. In this example, the base rate of the item of interest would be 0.70, or 70%. Hence, the base rate for the item of interest is calculated as:

*p* = *M*_UCT_ - *M*_Control_

where *p* is the proportion of subjects endorsing the item of interest. Random assignment and large sample sizes can reduce the likelihood of intergroup differences accounting for the mean difference. Wimbush and Dalton (1) suggest that the minimum group size for UCT should be 40 – 50 subjects. The present study utilized samples of approximately 700 (control condition = 703, UCT treatment condition = 699).

A control item is often included to act as a measure of UCT’s effectiveness. For instance, in the present study, one set in the UCT treatment condition, included the non-sensitive item:

“I have read (online or offline) material from any political group.”

This item was also included in the direct survey. Previous research suggests that there should be no significant differences between the reported base rates of the control item in the direct survey condition and the UCT condition (1).

Both groups received the same on-screen instructions, as below:

“This section of the questionnaire is designed to encourage honest reporting. You will be presented with a set of statements. You will not be asked to indicate which of the statements are true for you. You will only be asked to indicate how many of the statements are true for you. For example, in the following set:

I like the beach.

I have watched a play this month.

I have art on my wall.

I have a guitar.

I have a cactus.

If you 'like the beach' and 'watched a play this month' but the rest of the statements were not true for you, you would select '2' as your answer. Only the number you choose as your answer will be visible to researchers. You are not endorsing which statements are true, simply how many are true for you. Therefore, there is no way to identify which statements are true for you.

The control group only answered question sets containing items not included in our lone-actor terrorist codebook (such as the above example). These items were drawn from existing studies that have previously utilized UCT designs. Where additional control items were required, novel items were generated in the style and matching the general content of previously published items. Consideration was given to the likely base rates of the control items in order to design against ceiling effects. There were 25 sets of five non-sensitive items. Given random assignment, matched demographics, and large enough sample sizes, the base rates of control items are assumed to occur equally across the conditions. Hence, the difference between the mean number of statements endorsed by the control condition and the UCT condition is the proportion of participants in the UCT condition who endorse the additional item.

The UCT condition duplicated the control condition exactly, with the addition of one sensitive item per set. Sensitive items were drawn directly from the lone-actor terrorist codebook. Items were deemed sensitive if they asked the respondent to self-report past or present illegal, undesirable, or risky behavior, such as previous criminal convictions, being violent as a child, or engaging with terrorist propaganda. Items relating to family or close associates engaging in these sorts of behaviors were also deemed sensitive. However, items related to witnessing others, beyond family members and close associates, engaging in such behaviors, were not deemed sensitive. For example, consider the above control set, with the addition of a sensitive item below:

I like the beach.

I have watched a play this month.

I was violent as a child/adolescent.

I have art on my wall.

I have a guitar.

I have a cactus.

Hence participants are able to endorse the sensitive item without signaling so to researchers.

TABLE S1—*Base rate estimates of risk factors/indicators associated with violent extremism.*

To note, in the below table we provide the base rate estimates for all items asked in The Base Rate Survey (available on the OSF at <https://osf.io/gjx4q/>). Some of these items are not reported in the subsequent analyses as we did not have comparable data in the offending sample. However, these still may be of interest to others.

| Item Code | Item Question | % (n = 2108) |
| --- | --- | --- |
| AbuseHome | Did you grow up in an abusive home (e.g. mother was victim of domestic abuse), but were not a victim of abuse yourself? | 15.0% |
| Anger | Have you ever had difficulties managing your anger? | 31.5% |
| AngerEscalating | In the last year, has your anger been escalating? (i.e. getting more intense, or having outbursts of anger more frequently) | 9.2% |
| AwareGrievance | Have you ever known someone who has expressed extreme political, religious or social grievances? | 23.3% |
| AwareIdeology | Have you ever known someone who identified with an ideology that supports violent action? | 22.6% |
| ChronicStress | Are there any long-term sources of stress in your life? | 52.0% |
| Combat | Do you have armed combat experience? | 3.3% |
| CommunityViol | Does your community (e.g. friends, acquaintances, social group) disapprove of others in your community committing acts of violence? | 85.1% |
| Crisis | In the last year, have you experienced a period of crisis (e.g. Family loss/break up? Failure in educational career? Unemployment? Prison? Migration to new country | 33.6% |
| Degraded | In the last year, have you experienced being humiliated or degraded? | 22.8% |
| DiagNo | How many diagnosed mental disorders do you have? | 44.4% |
| DirectThreat | Has anyone ever communicated to you a direct threat of political violence? | 7.8% |
| Disrespect | In the last year, have you experienced being disrespected? | 58.6% |
| DropOut | In the last year, have you dropped out of school/university? | 2.8% |
| Exceptional | Do you have any exceptional academic achievement? | 36.9% |
| Expel | Were you ever thrown out of an educational environment as a child or adolescent (e.g. expelled from school)? | 5.9% |
| FamDeath | In the last year, has there been a death in your immediate family? | 27.1% |
| FamilyViol | Does your family disapprove of other family members committing acts of violence? | 89.0% |
| Financial | In the last year, have you experienced financial problems? | 49.3% |
| Harm | In the last year, have you experienced being harmed due to the negligence of someone else? | 9.8% |
| Helpless | In the last year, have you experienced being feeling helpless or victimised? | 30.0% |
| HouseReli | Were you raised in a household where one or more parent or guardian followed a religious practice? | 43.9% |
| IdeoIntense | If [IdeoViolence] yes, did their ideological beliefs intensify in the buildup (to a violent extremist act)? | 1.9% |
| IdeoLeftWing | If [AwareIdeology] yes, what was their ideology? [Left wing] | 3.6% |
| IdeoNationalist | If [AwareIdeology] yes, what was their ideology? [Nationalist] | 4.5% |
| IdeoOther | If [AwareIdeology] yes, what was their ideology? [Other] | 1.7% |
| IdeoReligious | If [AwareIdeology] yes, what was their ideology? [Religious] | 3.7% |
| IdeoRightWing | If [AwareIdeology] yes, what was their ideology? [Right wing] | 5.7% |
| IdeoSingleIssue | If [AwareIdeology] yes, what was their ideology? [Single issue] | 3.6% |
| IdeoViolence | If [AwareIdeology] yes, did they commit an act of extremist violence? | 3.7% |
| Ignore | In the last year, have you experienced being ignored or treated poorly by someone important to you? | 52.1% |
| Inflexible | Do you find it difficult to adapt to challenges or obstacles? | 42.4% |
| Interrupt | In the last year, has your work/progress towards an important goal been interrupted? | 33.4% |
| Isolated | Are you physically/socially isolated from others? (e.g. do you live alone or away from family, or feel lonely) | 42.4% |
| IsolatedT | Was this in the last year? | 37.9% |
| Letters | Have you witnessed anyone producing letters or public statements expressing their extremist beliefs or intent to commit political violence | 13.4% |
| LowControl | [Dichotomized from scale items] | 10.1% |
| MentalDisorder | Do you have a history of diagnosed mental illness? | 26.2% |
| MentalDisorderT | Was this in the last year? | 21.6% |
| MilExp | Do you have any previous military experience | 2.8% |
| MilitaryCurr | Are you currently in the military? | 0.3% |
| MilRej | Have you ever been rejected from the military? | 1.8% |
| MilYears | If yes, how many years military experience do you have? | 4.3 |
| NotCare | In the last year, have you experienced someone important demonstrating they do not care about you? | 36.4% |
| Obsess | Have you ever been obsessed with one specific event or phenomena to the extent that it consumed a significant amount of your attention? | 37.3% |
| ObsessT | Was this in the last year? | 21.9% |
| OverConfidence | [Dichotomized from scale items] | 20.3% |
| PersRel | In the last year, have you experienced problems with personal? relationships | 49.3% |
| Prejudice | In the last year, have experienced being the target of an act of prejudice or unfairness based on your religion, ethnicity, sexuality, gender or other personal characteristic? | 16.0% |
| Promise | In the last year, have you experienced being lied to or having a promise broken? | 53.4% |
| ProxChange | In the last year, have you experienced a significant and difficult change in your life circumstances (e.g., deployment, breakdown of a marriage, international relocation)? | 18.3% |
| PsychDistress | Have you experienced mental health issues short of a diagnosis of mental illness? | 53.8% |
| PsychDistressT | Was this in the last year? | 38.8% |
| RecentUnemploy | In the last year, have you lost your job? | 7.6% |
| RelCon | Have you undergone an experience of religious conversion at any time? | 13.5% |
| RelConT | Was this in the last year? | 0.8% |
| ReligChange | If [IdeoViolence = yes] did they change religions in the buildup (to a violent extremist act)? | 0.4% |
| ReligIntense | If [IdeoViolence] yes, did their religious beliefs intensify in the buildup (to a violent extremist act)?? | 0.5% |
| SocialIsolate | Do you isolate yourself from others intentionally? (e.g. do you purposefully avoid engaging with others, socially) | 23.2% |
| ThrillSeeking | [Dichotomized from scale items] | 14.0% |
| VerbState | Have you ever witnessed someone making verbal statements expressing their extremist beliefs or intent to commit extremist violence to a general audience? | 33.9% |
| Victim | In the last year, have you experienced being the victim of verbal or physical assault? | 20.2% |
| VictimBully | Were you a victim of bullying as a child or adolescent? | 50.9% |
| VictimOther | Were you a victim of violence other than domestic abuse or bullying as a child or adolescent? | 11.8% |
| ViolentAttitudes | [Dichotomized from a scale item 'It is OK to use violence to achieve my goals'] | 12.8% |
| WorkStress | In the last year, have you experienced a work-related loss or stressor? (e.g. demotion, disciplined, conflict)? | 29.9% |
| WorsePerf | In the last year, has your performance at work/school significantly worsened? | 16.3% |
| Item Code | Item Question | % (n = 706) |
| Control | Have you read (online or offline) material from any political group? | 56.1% |
| CloseAssociates | Are any of your family or close associates known to have been involved in political extremism? | 1.7% |
| CrimCon | Do you have any previous criminal convictions? | 2.5% |
| ExtremeBeliefs | Have you ever held extremist beliefs | 4.2% |
| ExtremeBeliefsT | Was this in the last year? | 2.3% |
| F2F | Have you ever interacted face-to-face with members of a political group who support the use of violence? | 7.2% |
| HurtOthers | Have you ever expressed a desire to hurt others? | 12.7% |
| HurtOthersT | Was this in the last year? | 7.4% |
| Imprison | Were you ever imprisoned | 0.4% |
| ImprisonAge | If yes, how old were you when you went to prison? | 27.3 years |
| ImprisonYears | If yes, how long were you imprisoned for? | 0.6 years |
| JuvArrest | Were you ever arrested as a child or adolescent? | 5.0% |
| OnlineExtreme | Have you searched for extremist content online? | 6.8% |
| OnlineExtremeT | Was this in the last year? | 3.7% |
| PerpAbuse | As an adult, have you ever physically hurt (punched, kicked, slapped etc.) or caused physical injury, to a romantic partner or family member? | 10.1% |
| PropagandaGroup | Have you ever read or consumed the literature or propaganda from a group who supports the use of violence to advance political goals? (e.g. Facebook pages, pamphlets, web pages, letters) | 19.5% |
| PropagandaLA | Have you ever consumed propaganda produced by lone actor terrorists, such as their manifestos or videos? | 18.7% |
| PropagandaOther | Have you ever read or consumed literature or materials about lone actor terrorists? | 11.9% |
| Recruit | Have you ever tried to recruit others to form a political group based on views that support the use of violence? | 0.1% |
| Rejected | Were you ever rejected from a political group? | 0.6% |
| SpouseInvolved | Is/Was your spouse/partner part of a political group that supports violent action? | 0.8% |
| Stockpile | Have you ever had access to a stockpile of weapons? | 3.3% |
| StockpileT | Was this in the last year? | 1.7% |
| SubAbuse | Do you have a history of substance abuse? | 9.5% |
| SupportChild | Did you ever need special care or attention as a child? (e.g. school support, short of involvement with social services or social care) | 8.1% |
| VerbFam | Has anyone in your family ever made verbal statements expressing extremist beliefs or their intent to commit extremist violence? | 4.2% |
| Violence | Have you ever committed an act of violence as an adult? | 6.5% |
| ViolenceT | Was this in the last year? | 0.8% |
| ViolentChild | Were you violent as a child or adolescent? (e.g. physical bullying, criminal violence) | 5.1% |
| VirtualInteract | Have you ever interacted virtually with members of a political group that supports violent action? | 10.9% |
| WiderGroup | Have you ever been a member of a political group that supports violent action? | 0.1% |
| WiderGroupActivism | Did you take part in high-risk political activism on behalf of that group? | 0.1% |

TABLE S2—*A comparison of lone-actor terrorists with a sample from the general population across propensity indicators.*

| Propensity indicators (non-sensitive) | General population  (*n* = 2,108) | Lone-actor terrorists  (*n* = 125) | Chi-square statistic | Std. Err | Lower bound 95% CI | Upper bound 95% CI |
| --- | --- | --- | --- | --- | --- | --- |
| Unemployed | 14.0% | 38.4%*** | 54.06 | 0.033 | -0.3091 | -0.1790 |
| Previous military experience | 2.8% | 22.4%*** | 119.18^a^ | 0.018 | -0.2306 | -0.1604 |
| Currently in the military | 0.3% | 4.0%*** | 33.23^a^ | 0.006 | -0.0498 | -0.0245 |
| Evidence of thrill-seeking behaviours | 14.0% | 29.6%*** | 22.51 | 0.033 | -0.2199 | -0.0913 |
| Low self-control | 10.1% | 36.0%*** | 77.98 | 0.029 | -0.3170 | -0.2018 |
| Diagnosed mental disorder | 26.2% | 40.8%** | 12.69 | 0.041 | -0.2258 | -0.0655 |
| University experience | 52.8%*** | 35.2% | 14.56 | 0.046 | 0.0854 | 0.2657 |
| Exceptional educational achievements | 36.9%*** | 16.8% | 20.68 | 0.044 | 0.1141 | 0.2871 |
| Grew up in an abusive home | 15.0%*** | 4.0% | 11.58 | 0.032 | 0.0466 | 0.1732 |
| Victim of bullying as a child/adolescent | 50.9%*** | 12.0% | 71.65 | 0.046 | 0.2993 | 0.4797 |
| Chronic stress | 52.0%*** | 31.2% | 20.51 | 0.046 | 0.1182 | 0.2986 |
| Children | 30.5%* | 20.8% | 5.25 | 0.042 | 0.0139 | 0.1792 |
| Victim of violence other than bullying/DV | 11.8%* | 4.8% | 5.68 | 0.029 | 0.0124 | 0.1269 |
| Expelled from any educational institution | 5.9% | 4.0% |  |  |  |  |
| Obsessed with an event or phenomenon | 37.3% | 28.8% |  |  |  |  |
| Rejected from the military | 1.8% | 3.2% |  |  |  |  |
| Single | 34.7% | 42.4% |  |  |  |  |
| Grew up in a religious household | 43.9% | 36.0% |  |  |  |  |
| Underwent a religious conversion | 13.5% | 18.4% |  |  |  |  |
| Over-confidence/self-aggrandizement | 20.3% | 16.8% |  |  |  |  |

TABLE S3—*A comparison of lone-actor terrorists with a sample from the general population across situation indicators.*

| Situational indicators | | General population  (*n* = 2,108) | Lone-actor terrorists  (*n* = 125) | Chi square statistic | Std Error | Lower bound 95% CI | Upper bound 95% CI |
| --- | --- | --- | --- | --- | --- | --- | --- |
| Proximal crisis | | 33.6% | 53.6%*** | 20.86 | 0.021 | -0.5010 | -0.4200 |
| Dropped out of school/university | | 2.8% | 12.8%*** | 37.19 | 0.028 | -0.3156 | -0.2043 |
| Escalating anger | | 9.20% | 35.3%*** | 83.75 | 0.034 | -0.1389 | -0.0053 |
| Experienced prejudice/injustice | | 16.0% | 23.2%* | 4.48 | 0.040 | 0.1198 | 0.2780 |
| Family death | | 27.1%*** | 7.2% | 24.27 | 0.043 | 0.1221 | 0.2908 |
| Interrupted in pursuit of a proximate goal | | 33.4%*** | 12.8% | 23.00 | 0.046 | 0.4355 | 0.6159 |
| Had a promise broken | | 53.4%*** | 0.8% | 130.45 | 0.046 | 0.2804 | 0.4593 |
| Experienced being disrespected | | 58.6%*** | 21.6% | 65.69 | 0.046 | 0.3351 | 0.5156 |
| Ignored by someone important to them | | 52.1%*** | 9.6% | 85.40 | 0.044 | 0.1738 | 0.3459 |
| Not cared for by someone important | | 36.4%*** | 10.4% | 35.06 | 0.042 | 0.0980 | 0.2617 |
| Felt like a helpless victim | | 30.0%*** | 12.0% | 18.54 | 0.046 | 0.1307 | 0.3110 |
| Problematic personal relationships | | 49.3%*** | 27.2% | 23.06 | 0.046 | 0.1392 | 0.3195 |
| Financial problems | | 49.3%*** | 26.4% | 24.87 | 0.027 | 0.0374 | 0.1421 |
| Harmed by the negligence of someone else | | 9.8%** | 0.8% | 11.29 | 0.037 | 0.0103 | 0.1539 |
| Victim of physical/verbal assault | | 20.2%* | 12.0% | 5.02 | 0.021 | -0.5010 | -0.4200 |
| Recently became unemployed | | 7.6% | 29.6% |  |  |  |  |
| Experienced being degraded | | 22.8% | 16.8% |  |  |  |  |
| Proximate life change | | 18.3% | 11.3% |  |  |  |  |
| Situational indicators | Direct sample  (*n* = 706)  Life-time prevalence | In the last year | Lone-actor terrorists  (*n* = 125) | Chi square statistic | Std Error | Lower bound 95% CI | Upper bound 95% CI |
| Expressed a desire to hurt others | 12.7% | 7.4% | 64.0%*** | 171.43 | 0.039 | 0.4358 | 0.5892 |
| Committed an act of violence | 6.5% | 0.8% | 41.6%*** | 125.67 | 0.031 | 0.2895 | 0.4122 |
| Access to a stockpile of weapons | 3.3% | 1.7% | 54.8%*** | 287.47 | 0.030 | 0.4521 | 0.5709 |

***** p <.000, ** p <.00, *p <.05

TABLE S4—*A comparison of lone-actor terrorists with a sample from the general population across exposure indicators.*

| Exposure indicators | Direct sample  (*n* = 706) | Lone-actor terrorists  (*n* = 125) | Chi square statistic | Std error | Lower bound 95% CI | Upper bound 95% CI |
| --- | --- | --- | --- | --- | --- | --- |
| Joined a wider group | 0.1% | 31.2%*** | 223.58 | 0.021 | 0.2699 | 0.3513 |
| Close associates involved in violent/ extremist action | 1.7% | 25.6%*** | 120.98 | 0.022 | 0.1964 | 0.2816 |
| Face-to-face interactions with extremists | 7.2% | 39.2%*** | 102.58 | 0.032 | 0.2579 | 0.3816 |
| Virtual interactions with extremists | 10.9% | 31.2%*** | 36.41 | 0.033 | 0.0672 | 0.1946 |
| Attempted to recruit others to join/form a wider group | 0.1% | 19.2%*** | 132.19^a^ | 0.016 | -0.0645 | -0.0006 |
| Rejected from a political group | 0.6% | 8.8%*** | 40.62^a^ | 0.013 | 0.0570 | 0.1077 |
| Engaged with propaganda of wider group | 19.5% | 62.4%*** | 101.38^a^ | 0.043 | 0.3451 | 0.5120 |
| Spouse involved in wider movement | 0.8% | 5.6%** | 15.56^a^ | 0.012 | 0.0239 | 0.0711 |
| Engaged with propaganda by lone-actor terrorists | 18.7% | 26.4%* | 3.96 | 0.032 | -0.0142 | 0.1122 |
| Engaged with materials about other lone-actor terrorists | 11.9% | 16.8% |  |  |  |  |

***** p <.000, ** p <.01, *p <.05, Fisher’s exact ^a^

*Discussion of the UCT – What Went Wrong?*

Contrary to much previous research, the results suggest that the UCT protocol did not elicit higher base rates than the direct survey protocol. This has been reported previously (2-5). Sometimes this is presented as evidence that subjects may overreport items perceived to be socially desirable, despite being sensitive (5). This could account somewhat for the present findings. For instance, considering the control item, engaging with mainstream politics may be deemed socially desirable by some, and hence subjects may overreport whether they have engaged with the materials of any political group in a direct survey. This may explain why 56.1% of respondents endorsed this statement in the conventional survey and just 27.4% did so in the UCT protocol. However, Starosta and Earleywine (5) report lower base rate estimates for socially undesirable items too. This is interpreted as evidence that participants may also overreport socially undesirable items. However, this may in fact be a deflation effect.

In a number of instances, participants endorsed significantly fewer statements overall (resulting in negative estimates of the overall base rates) in the UCT condition. This may be a deflation effect given the nature of the items (relating to terrorism and extremism), the nature of the sample, and the delivery mode of the survey. Prolific’s subject pool are experienced survey respondents whose perceptions of their own anonymity may be different to the more traditional, offline subject, i.e. an undergraduate student participating in a pencil-and-paper survey at a university. First, Prolific users are operating online, which negates the need for face-to-face contact. This may increase a user’s perception of their own anonymity in itself, as previously discussed. Second, these users are assured of their anonymity by Prolific, as well as by researchers utilizing the platform. More importantly, the majority of these users regularly use Prolific and so may have greater trust, through lived experience, in assurances of their anonymity. Hence, the UCT manipulation, under the present study conditions, may not be necessary. In fact, it may have had a countereffect, where we see deflation.

One explanation would be that users may have been suspicious of the UCT protocol and the relevance of the innocuous items to the risk assessment of terrorists (anecdotally, we did receive some communications expressing such concerns). Moreover, many of these users often participate in a wide range of research and are exposed to a plethora of questions and content, including those sensitive in nature. Upon registering for the service, Prolific asks users pre-screening questions about their criminal histories and mental health, for example. If users are deterred by the level of disclosure required, perhaps they may not complete registration. Hence the nature of the Prolific sample may in fact facilitate the use of direct questioning methods. Given these findings, we employed the direct survey data in comparison with the lone-actor terrorists sample, as we believed it to be the more accurate estimate of the base rates of the items of interest.

The conditions under which some indirect questioning protocols result in more truthful answers have been explored empirically (6). Moreover, previous studies utilizing UCT have reported deflation effects. Zigerell (7) state misreporting is more common when 1) the items are very socially undesirable, 2) more respondents desire being associated or disassociated with the item, 3) respondents associate with many, or few, of the control items. In the present study, care was taken to design against very high, or very low variance (and examined post data-collection), in an attempt to control for ceiling or near-ceiling effects. However, the remaining points may be valid given the nature of the sensitive items investigated here. Lastly, direct questioning has a number of advantages over indirect questioning, particularly considering the advantages of obtaining participant-level estimates over aggregated group-level base rates, and so these findings may in fact be useful for future research, particularly in terrorism studies.

**References**

1. Wimbush JC, Dalton, DR. Base rate for employee theft: Convergence of multiple methods. J Appl Psychol 1997;82(5):756–63. doi: 10.1037/0021-9010.82.5.756.
2. Ahart AM, Sackett PR. A new method of examining relationships between individual difference measures and sensitive behavior criteria: evaluating the unmatched count technique. Organ Res Methods 2004;7(1):101–14. doi: 10.1177/1094428103259557.
3. Biemer P, Brown G. Model-based estimation of drug use prevalence using item count data. J Off Stat 2005;21(2):287–308.
4. Biemer P, Jordan B, Hubbard M, Wright D. A test of the item count methodology for estimating cocaine use prevalence. In: Kennet J, Gfroerer J, editors. Evaluating and improving methods used in the National Survey on Drug Use and Health. Rockville, MD: Substance Abuse and Mental Health Services Administration, 2005;149–74.
5. Starosta AJ, Earleywine M. Assessing base rates of sexual behavior using the unmatched count technique. Health Psychol Behav Med: Open Access Journal 2014;2(1):198–-210. doi: 10.1080/21642850.2014.886957.
6. John LK, Loewenstein G, Acquisti A, Vosgerau J. When and why randomized response techniques (fail to) elicit the truth. Organ Behav Hum Decis Process 2018;148:101–23. doi: [10.1016/j.obhdp.2018.07.004/](https://doi.org/10.1016/j.obhdp.2018.07.004/).
7. Zigerell LJ. You wouldn't like me when I'm angry: list experiment misreporting. Soc Sci Q 2011;92(2):552–62.
